# Endoscopic management of a post-gunshot biliary injury in a conflict zone: a case report from Sudan

**DOI:** 10.1093/jscr/rjaf730

**Published:** 2025-09-13

**Authors:** Abderrahaim Ali Dabora, Hala Ibrahim Abdalla, Ahmed Rafei, Abdalla Bedab, Mohammed Ganim, Fayad Mohamed Alzain Mohamed, Maha Omer, Abdelmoneim Eltayeb Abdo

**Affiliations:** Consultant of Hepatobiliary and Pancreatic Surgery, National Center for Gastrointestinal and Liver Diseases, Khartoum, Sudan; Consultant of Physician and Gastroenterologist, National Center for Gastrointestinal and Liver Diseases, Khartoum, Sudan; Department of Research, National Center for Gastrointestinal and Liver Diseases, Khartoum, Sudan; Department of Research, National Center for Gastrointestinal and Liver Diseases, Khartoum, Sudan; Department of Research, National Center for Gastrointestinal and Liver Diseases, Khartoum, Sudan; Department of Research, National Center for Gastrointestinal and Liver Diseases, Khartoum, Sudan; Consultant Radiologist, National Center for Gastrointestinal and Liver Diseases, Khartoum, Sudan; Consultant of Physician and Gastroenterologist, National Center for Gastrointestinal and Liver Diseases, Khartoum, Sudan

**Keywords:** traumatic bile leak, gunshot injury, ERCP, biliary fistula, liver trauma, Sudan

## Abstract

Traumatic biliary injuries are rare but serious complications following abdominal trauma, often presenting significant diagnostic and therapeutic challenges, particularly in resource-limited, conflict-affected settings. We report the case of a 31-year-old male soldier who sustained gunshot injuries to the abdomen and left hand during armed conflict. He was hemodynamically unstable on presentation and underwent emergency exploratory surgery, during which liver injury was identified and bleeding controlled. Seven days postoperatively, a biliary fistula developed, confirmed by magnetic resonance cholangiopancreatography (MRCP). The patient was referred for endoscopic management, where endoscopic retrograde cholangiopancreatography (ERCP) with sphincterotomy and biliary stenting was successfully performed. Follow-up ERCP after 6 months showed complete resolution of the biliary fistula, and the stent was safely removed. This case highlights the critical role of ERCP in managing traumatic bile leaks, especially in war zones where surgical interventions may be limited. Endoscopic therapy offers a less invasive and highly effective alternative for treating biliary complications in complex scenarios.

## Introduction

Biliary leaks result from disruptions in the biliary system, most commonly due to iatrogenic injury, trauma, or hepatobiliary surgery [1]. Traumatic biliary injury is a rare condition, accounting for only 1%–3.5% of all abdominal injuries [2]. These injuries may occur due to penetrating trauma, such as gunshot or stab wounds, or blunt trauma from motor vehicle or motorcycle accidents [1]. If left untreated, biliary disruption can lead to bile peritonitis, potentially resulting in intrahepatic biloma and intraperitoneal bile leakage [3].

Traumatic biliary injury presents a significant clinical challenge for surgeons and phyisicans, as it is frequently associated with major organ damage [4]. The role of endoscopic management using endoscopic retrograde cholangiopancreatography (ERCP) has been investigated in a limited number of studies, which have demonstrated its efficacy in both diagnosis and treatment [5]. ERCP has shown a success rate of 89% in managing biliary complications when combined with biliary stenting and sphincterotomy. However, data on its outcomes specifically in the context of traumatic bile leaks remain limited [5, 6].

We present a case of a post-gunshot biliary leak managed effectively with ERCP in a conflict zone with limited surgical resources.

## Case presentation

A 31-year-old male soldier sustained gunshot wounds to the abdomen and left hand during armed conflict. He had no prior medical or surgical history. On arrival, he was hemodynamically unstable and received two liters of blood before undergoing emergency surgery.

Intraoperatively, a transected left brachial artery was repaired using a long saphenous vein bypass. The deep vein was ligated. Upon abdominal exploration through a midline laparotomy, active bleeding was identified from a laceration in the left lobe of the liver, specifically involving segments III and IV. Hemorrhage control was achieved through a combination of direct pressure, electrocautery, and application of hemostatic agents. No formal hepatic resection was required. The bleeding source appeared to be from a superficial parenchymal injury without major vascular involvement. After achieving hemostasis, the surgical team placed abdominal drains to monitor for potential bile or blood leakage.

Postoperatively, the patient’s laboratory results were unremarkable: total bilirubin was 1.0 mg/dL, direct bilirubin 0.2 mg/dL, prothrombin time 17.2 s, INR 1.16, and hemoglobin 12.7 g/dL.

**Figure 1:**
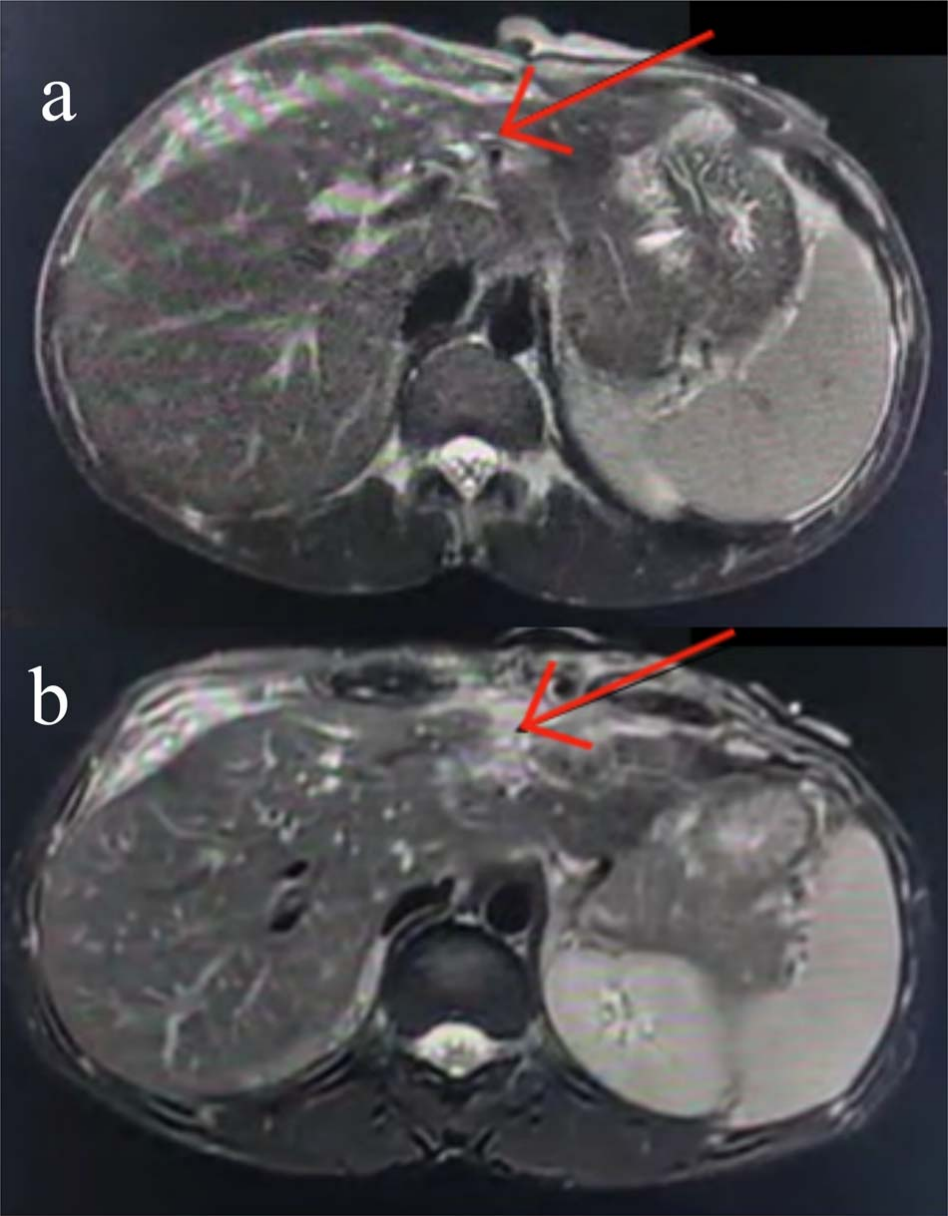
Axial contrast-enhanced abdominal CT images. (a) Subtle injury to the intrahepatic biliary radicals in the left hepatic lobe. (b) Linear laceration on the anterior surface of the liver.

Following stabilization, an abdominal ultrasound showed minimal free intraperitoneal fluid, and a contrast-enhanced computed tomography (CT) scan revealed lacerations in liver segments III and IV with a fluid pocket extending to the abdominal wall, suggestive of bile leakage. A sinus tract connecting the liver to the skin was also seen, along with mild intrahepatic biliary dilation (Fig. 1).

**Figure 2:**
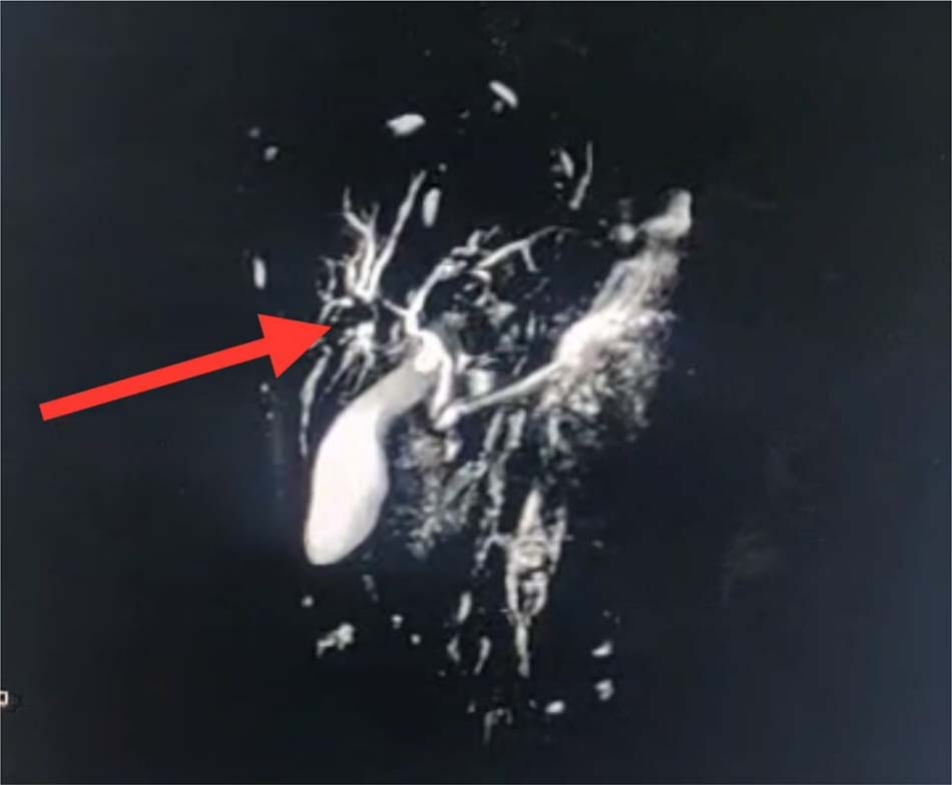
A coronal MRCP image highlighting disruption at the level of the left intrahepatic biliary radicals, suggestive of a biliary leak. The discontinuity is evident at the confluence of the left hepatic duct (arrow), corresponding with the previously identified liver laceration. The extrahepatic biliary tree appears normal with no signs of obstruction or dilatation.

Seven days post-op, bile leakage from the surgical site raised suspicion of a biliary fistula. MRCP confirmed a hepatic parenchymal defect in communication with the biliary system and an external draining tract through the abdominal wall. A 6 × 1 cm subcapsular collection was also noted (Fig. 2).

**Figure 3:**
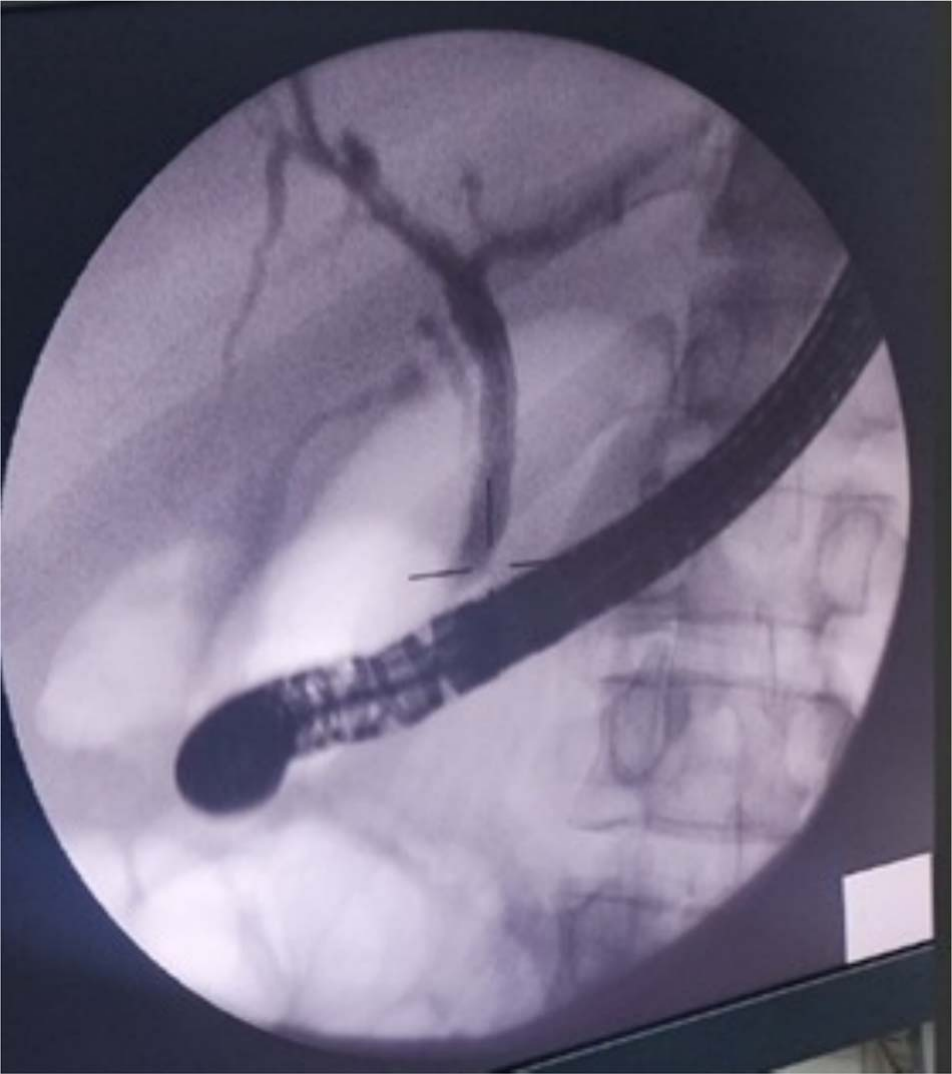
ERCP image showing a normal biliary system. A biliary stent was successfully placed in the right hepatic duct.

The case was referred to our unit and the decision was made to proceed with ERCP and biliary stenting. During the procedure, the common bile duct and intrahepatic ducts appeared normal. A sphincterotomy was performed, and an 8 Fr, 10 cm stent was placed in the right hepatic duct (Fig. 3), achieving good bile reflux. No obvious bile leak was detected.

The patient was treated with biliary stenting, and the fistula was monitored. The percutaneous leak stopped, ERCP and stent removal was planned after 6 months. The patient showed no signs of infection or sepsis throughout the post ERCP e course.

Around 6 months later, the patient underwent a follow-up ERCP. The biliary system was intact, with no contrast leak seen, and the previous stent was removed without complications. The biliary fistula was successfully resolved.

## Discussion

Abdominal injuries which, account for 12% of trauma incidents [7], are classified as blunt or penetrating, with the latter often requiring surgical intervention. Although surgery remains the primary treatment modality, endoscopic techniques are increasingly employed as adjuncts or alternatives. While trauma-related cases constitute a small proportion of endoscopic interventions, established endoscopic approaches for managing iatrogenic biliary, pancreatic, and bowel injuries can be effectively adapted for the treatment of traumatic injuries [8].

The ongoing war in Sudan has led to increased incidence of penetrating injuries, including liver trauma with biliary leaks [9]. In such resource-limited environments, nonoperative strategies like ERCP are crucial when surgical options are restricted.

Symptoms of traumatic biliary injury are often nonspecific and may include fever, nausea, vomiting, right upper quadrant pain, or jaundice, resembling those of iatrogenic biliary injury. If left untreated, biliary leaks can result in uncontained intra-abdominal bile spillage, leading to peritonitis, or form organized collections (bilomas). Bilious output from surgically placed percutaneous drains or a surgical incision site should be considered indicative of biliary injury following abdominal exploration [8]. In cases of blunt or penetrating liver injuries, maintaining a high index of suspicion for biliary leakage is crucial. Studies indicate that only 25% of iatrogenic biliary injuries are detected intraoperatively. If clinical suspicion persists, abdominal imaging is warranted [10].

ERCP remains the gold standard for diagnosing and treating biliary leaks, providing both visualization and therapeutic options in a single procedure [11]. Retrograde cholangiography categorizes bile leaks into low-grade and high-grade based on the timing of intrahepatic opacification. High-grade leaks are detected before opacification, whereas low-grade leaks become apparent only after intrahepatic biliary radicals are visualized. The choice of endoscopic management depends on leak severity, with biliary stenting and sphincterotomy achieving a 97% resolution rate in high-grade leaks. In contrast, sphincterotomy alone resulted in a 91% resolution rate for low-grade leaks [12].

Current evidence for endoscopic management of traumatic biliary injuries is primarily based on smaller retrospective case series. In one study, 10 patients with traumatic bile leaks underwent ERCP, with five cases resulting from gunshot wounds and five from blunt trauma. All patients received biliary stents, and two underwent sphincterotomy, yielding a 90% resolution rate [13]. Another series reported a 100% resolution rate among 14 patients who underwent ERCP with sphincterotomy and biliary stent placement, with blunt trauma accounting for eight of the cases [1]. Comparable favorable outcomes have been documented in smaller case series, reinforcing the role of ERCP as an effective intervention for traumatic biliary injuries [14, 15].

The successful endoscopic management of this patient’s traumatic biliary leak underscores the feasibility of ERCP in complex trauma cases. In conflict settings like Sudan, where access to surgical facilities may be limited, ERCP provides a valuable alternative for managing biliary leaks, reducing the need for invasive procedures.

## References

[1] Spinn MP, Patel MK, Cotton BA, et al. Successful endoscopic therapy of traumatic bile leaks. Case Rep Gastroenterol 2013;7:56–62. 10.1159/000346570 23525187 PMC3604865

[2] Erkan M, Bilge O, Ozden I, et al. Definitive treatment of traumatic biliary injuries. Ulus Travma Acil Cerrahi Derg 2004;10:221–5. 15497059

[3] Al Humayed SM . Endoscopic treatment of biliary leak following gunshot injury: a case report. Saudi J Med Med Sci 2018;6:112–4. 10.4103/sjmms.sjmms_121_16 30787832 PMC6196712

[4] Wahaibi AA, Alnaamani K, Alkindi A, et al. A novel endoscopic treatment of major bile duct leak. Int J Surg Case Rep 2014;5:189–92. 10.1016/j.ijscr.2014.01.017 24636979 PMC3980414

[5] Singh V, Narasimhan KL, Verma GR, et al. Endoscopic management of traumatic hepatobiliary injuries. J Gastroenterol Hepatol 2007;22:1205–9. 10.1111/j.1440-1746.2006.04780.x 17688661

[6] de Reuver PR, Rauws EA, Vermeulen M, et al. Endoscopic treatment of post-surgical bile duct injuries: long term outcome and predictors of success. Gut 2007;56:1599–605. 10.1136/gut.2007.123596 17595232 PMC2095661

[7] Gad MA, Saber A, Farrag S, et al. Incidence, patterns, and factors predicting mortality of abdominal injuries in trauma patients. N Am J Med Sci 2012;4:129–34. 10.4103/1947-2714.93889 22454826 PMC3309620

[8] Sealock RJ, Othman M, Das K. Endoscopic diagnosis and management of gastrointestinal trauma. Clin Gastroenterol Hepatol 2021;19:14–23. 10.1016/j.cgh.2019.09.048 31605872

[9] UNHCR. Sudan Crisis Explained [Internet]. UNHCR . 2024. Available from: https://www.unrefugees.org/news/sudan-crisis-explained/

[10] Stewart L . Iatrogenic biliary injuries: identification, classification, and management. Surg Clin North Am 2014;94:297–310. 10.1016/j.suc.2014.01.008 24679422

[11] Goble SR, Abdallah M, Rosenberg C, et al. Endoscopic management of pancreaticobiliary injuries: a level 1 US trauma center experience. Ochsner J 2024;24:184–91. 10.31486/toj.24.0040 39280866 PMC11398632

[12] Sandha GS, Bourke MJ, Haber GB, et al. Endoscopic therapy for bile leak based on a new classification: results in 207 patients. Gastrointest Endosc 2004;60:567–74. 10.1016/s0016-5107(04)01892-9 15472680

[13] Bridges A, Wilcox CM, Varadarajulu S. Endoscopic management of traumatic bile leaks. Gastrointest Endosc 2007;65:1081–5. 10.1016/j.gie.2006.11.038 17531646

[14] Sugiyama M, Atomi Y, Matsuoka T, et al. Endoscopic biliary stenting for treatment of persistent biliary fistula after blunt hepatic injury. Gastrointest Endosc 2000;51:42–4. 10.1016/s0016-5107(00)70385-3 10625794

[15] Lubezky N, Konikoff FM, Rosin D, et al. Endoscopic sphincterotomy and temporary internal stenting for bile leaks following complex hepatic trauma. Br J Surg 2006;93:78–81. 10.1002/bjs.5195 16315338

